# *In Vitro* comparison of ^213^Bi- and ^177^Lu-radiation for peptide receptor radionuclide therapy

**DOI:** 10.1371/journal.pone.0181473

**Published:** 2017-07-21

**Authors:** Ho Sze Chan, Erik de Blois, Alfred Morgenstern, Frank Bruchertseifer, Marion de Jong, Wouter Breeman, Mark Konijnenberg

**Affiliations:** 1 Department of Radiology and Nuclear Medicine, Erasmus MC, Rotterdam, The Netherlands; 2 European Commission, Joint Research Centre, Institute for Transuranium Elements (ITU), Karlsruhe, Germany; Brandeis University, UNITED STATES

## Abstract

**Background:**

Absorbed doses for α-emitters are different from those for β-emitters, as the high linear energy transfer (LET) nature of α-particles results in a very dense energy deposition over a relatively short path length near the point of emission. This highly localized and therefore high energy deposition can lead to enhanced cell-killing effects at absorbed doses that are non-lethal in low-LET type of exposure. Affinities of DOTA-DPhe^1^-Tyr^3^-octreotate (DOTATATE), ^115^In-DOTATATE, ^175^Lu-DOTATATE and ^209^Bi-DOTATATE were determined in the K562-SST2 cell line. Two other cell lines were used for radiation response assessment; BON and CA20948, with a low and high expression of somatostatin receptors, respectively. Cellular uptake kinetics of ^111^In-DOTATATE were determined in CA20948 cells. CA20948 and BON were irradiated with ^137^Cs, ^177^Lu-DTPA, ^177^Lu-DOTATATE, ^213^Bi-DTPA and ^213^Bi-DOTATATE. Absorbed doses were calculated using the MIRDcell dosimetry method for the specific binding and a Monte Carlo model of a cylindrical 6-well plate geometry for the exposure by the radioactive incubation medium. Absorbed doses were compared to conventional irradiation of cells with ^137^Cs and the relative biological effect (RBE) at 10% survival was calculated.

**Results:**

IC_50_ of (labelled) DOTATATE was in the nM range. Absorbed doses up to 7 Gy were obtained by 5.2 MBq ^213^Bi-DOTATATE, in majority the dose was caused by α-particle radiation. Cellular internalization determined with ^111^In-DOTATATE showed a linear relation with incubation time. Cell survival after exposure of ^213^Bi-DTPA and ^213^Bi-DOTATATE to BON or CA20948 cells showed a linear-exponential relation with the absorbed dose, confirming the high LET character of ^213^Bi. The survival of CA20948 after exposure to ^177^Lu-DOTATATE and the reference ^137^Cs irradiation showed the typical curvature of the linear-quadratic model. 10% Cell survival of CA20948 was reached at 3 Gy with ^213^Bi-DOTATATE, a factor 6 lower than the 18 Gy found for ^177^Lu-DOTATATE and also below the 5 Gy after ^137^Cs external exposure.

**Conclusion:**

^213^Bi-DTPA and ^213^Bi-DOTATATE lead to a factor 6 advantage in cell killing compared to ^177^Lu-DOTATATE. The RBE at 10% survival by ^213^Bi-ligand compared to ^137^Cs was 2.0 whereas the RBE for ^177^Lu-DOTATATE was 0.3 in the CA20948 in vitro model.

## Introduction

The receptor-mediated endocytosis pathway is one of the main pathways to deliver biomolecules in cells. Peptide receptor radionuclide therapy (PRRT) uses this process to deliver cytotoxic dose by the emission of β-particles to neuroendocrine tumours (NET). Somatostatin peptide analogues, such as DOTA-DPhe^1^-Tyr^3^-octreotide (DOTATOC) and DOTA-DPhe^1^-Tyr^3^-octreotate (DOTATATE), are the most common delivery systems for treatment of NET. By radiolabelling these analogues with β-emitting radionuclide such as ^90^Y (T_1/2_ = 64.1 h) or ^177^Lu (T_1/2_ = 6.6 d), high radiation doses can be delivered to tumour cells, causing mostly single-strand breaks (SSB) in the DNA of the tumour cells. Dependent on the number of SSB, cells can undergo cell arrest, with either activation of the cellular repair mechanism for repair or apoptosis as a consequence [1]. Combination of several repairable SSB lesions may lead to additional cell kill.

α-Emitters (e.g. ^213^Bi, T_1/2_ = 46 min;^225^Ac, T_1/2_ = 9.9 d; ^211^At, T_1/2_ = 7.2 h) are increasingly used for targeted alpha therapy (TAT) because of their emission of high linear energy transfer (LET) particles with a relative short path length. Labelled ^213^Bi-peptides have already been proven to be promising in PRRT with NETs in preclinical as well in clinical studies [2–5]. α-Emitters emit high LET particles, causing double-strand breaks (DSB) in DNA when targeted to the tumour cells [6]. Therefore, the cytotoxic property in cells is found to be greater for α-emitters than for β-emitters [6, 7].

The cytotoxic response of the cells is related to the absorbed dose delivered to the cells. Several studies have been investigating the absorbed dose caused in cells by α-emitters [8–10]. Those studies showed the challenge involved in describing dose-related survival in cells with α-particles radiation. Huang and co-workers distinguished three clear differences in cell dosimetry calculations for α-emitters compared to β-emitters or to external beam therapy; 1) short path length, 2) small target volume and 3) non-uniform distribution of radionuclides [11]. For β-emitters and external γ-beams, hundreds to thousands of ionizations are required for a cell-killing effect, whereas using α-emitters, this can be reached with 4–10 ionizations. Due to the low number of ionizations, leading to large variations in the number of α-particle tracks traversing the cells, the validity of the mean absorbed dose which assumes Poisson statistics, was not always given for α-emitters [12]. Moreover, variability in experiments strongly influenced the calculated absorbed dose, for example the models in which the absorbed dose was calculated; single cells, clusters of cells or whole organs. Furthermore, inhomogeneous uptake can also influence the calculated absorbed dose. The dose limits for α-emitters showed a high model dependence for selected survival endpoints, and therefore, the relative biological effect (RBE) should be considered within the same model and using the same endpoint. As mentioned, the calculation of the absorbed dose in vitro for α-emitters can be quite complicated. Many studies only mention the radioactivity administered to the cells instead of using absorbed dose. Therefore, the effective cytotoxic properties of α-emitters as published cannot easily be compared to each other on an absorbed dose level.

In this study we calculated the average absorbed dose delivered to single cells using non-specific and receptor-specific binding absorbed dose calculation methods. The non-specific binding method describes the homogeneous irradiation from medium without specific binding of labelled peptide to the receptors on the cell, whereas the specific binding method describes the specific binding of the labelled peptide to the receptor on the cell and the addition of homogeneous irradiation from medium to the cells. Affinity studies in K562-SSTR_2_ (transgenic human erythroleukemic cells transfected with somatostatin receptor subtype 2 (SSTR_2_)) cells were performed to determine the IC_50_ of DOTATATE, ^115^In-DOTATATE, ^175^Lu-DOTATATE, and ^209^Bi-DOTATATE. An internalization assay with CA20948 (rat pancreatic tumour) cell using ^111^In-DOTATATE was performed to obtain information of the kinetics of cell uptake. Based on results obtained from cell uptake, small-scale dosimetry calculations were performed to provide the additional absorbed dose caused by specific binding to the cells to obtain the correlation between absorbed dose and cell survival. We evaluated the RBE at the absorbed dose of 10% survival (*D*_10_) of ^213^Bi-DTPA, ^213^Bi-DOTATATE, ^177^Lu-DTPA, ^177^Lu-DOTATATE and external radiation using ^137^Cs using two different cell lines; CA20948 [13] with high and BON (human carcinoid) with low SSTR_2_ expression [14]. The aim of the study was to compare the effective cytotoxic properties of different irradiation methods i.e. external photon irradiation and targeted radionuclide therapy in the same study.

## Materials and methods

All chemicals were purchased from Sigma Aldrich, culture media for cell culture and in vitro assays were purchased from Gibco, Life Technologies, unless otherwise indicated.

### Cell culture

K562-SST_2_ is a human erythroleukemic transgenic cell line with an over expression of SSTR_2_ [15] and was a gift of prof. L. Hofland and prof. P.M. van Hagen (Erasmus MC, Rotterdam, the Netherlands). Cells were cultured in RPMI 1640 supplemented with 10% of fetal calf serum (Gibco, Life Technologies). CA20948 tumour cells [16] were cultured in DMEM supplemented with 10% fetal calf serum. Human carcinoid BON cells (American Tissue Culture Collection, Wesel, Germany) were cultured in F12-DMEM. The medium was supplemented with 10% fetal calf serum. All cells were cultured in T175 tissue culture flasks at 37°C in a humidified atmosphere of 5% CO_2_.

### Radiolabelling and radioiodination of peptides

^111^In-DOTATATE with a molar activity (MA) of 15 MBq/nmol was prepared by incubation of 15 MBq ^111^InCl_3_ (T_1/2_ = 2.8 d, γ of 171 and 245 keV, Covidien), DOTATATE (Mw 1436 g/mol, Biosynthema, St. Louis, MO, USA), sodium acetate 2.5 M, ethanol and a mixture of gentisic acid/ascorbic acid 50 mM in a volume of 140 μL at 80°C for 20 min. After incubation, 5 μL 10 mM DTPA (diethylenetriaminepentaacetic acid) was added to stop the reaction and to chelate any “unbound” or “free” ^111^In. MA is expressed in MBq per nmol peptide.

^177^Lu-DOTATATE (at MA 53 MBq/nmol) was prepared under the same labelling conditions as ^111^In-DOTATATE described above. ^177^LuCl_3_ was purchased from IDB Holland B.V (Baarle Nassau, the Netherlands).

For the labelling of ^213^Bi, a ^225^Ac/^213^Bi generator (≤ 222 MBq) was eluted with a fixed elution volume of 600 μL 0.1M/0.1M NaI/HCl [17]. The ^213^Bi containing elution was added to a mixture of 7 nmol DOTATATE, 60 μL TRIS 2M, 1.85 μL ascorbic acid 20% and MQ (final volume 800 μL). The reaction was performed at 95°C for 5 min and cooled on ice for 2 min afterwards. 5 μL of DTPA 10 mM was added to stop the labelling and chelate “unbound”/”free” ^213^Bi [18].

To determine the incorporation of the radioactivity, ITLC (Instant Thin-Layer Chromatography) was performed after each labelling. HPLC (High Performance Liquid Chromatography) was performed to determine the radiochemical purity (RCP) of the radiopeptides as described by the Blois et al. [19]. RCP of the labelled peptide was expressed as percentage of intact labelled peptide of interest versus of all other radioactive detectable compounds.

Tyr^3^-octreotide (Mw = 1034 g/mol, Biosynthema, St. Louis, MO, USA) was used to prepare ^125^I-Tyr^3^-octreotide using chloramine-T as described elsewhere [20]. Analysis and purification of the radioiodinated peptide were performed using HPLC as described by de Blois et al [19].

### Labelling of non-radio-peptide

The labelling of ^115^In-DOTATATE was performed by addition of ^115^InHNO_3_ (ICP standard, 1 g/L) to DOTATATE at a molar ratio of 5:1. The pH was adjusted to a pH of 4 by adding sodium acetate (2.5 M) and the labelling mixture was heated for 30 min at 80°C. Quality control was performed using HPLC as described previously [19]. UV- detection was performed at 278 nm. Under these conditions, DOTATATE was fully incorporated with ^115^In. After quality control the labelled peptide was purified and collected using the same HPLC method. The concentration of the labelled peptide was determined by UV spectrophotometer at 278 nm. This procedure was also performed for the labelling of ^175^Lu-DOTATATE and ^209^Bi-DOTATATE.

### IC_50_

IC_50_ values of DOTATATE, ^115^In-DOTATATE, ^175^Lu-DOTATATE or ^209^Bi-DOTATATE were determined using K562-SST_2_ membranes [21]. In short, cell membranes were isolated as described by Reubi [22]. Freshly dispersed membrane preparations (corresponding to 25 μg protein) were incubated at room temperature for 60 min with ^125^I-Tyr^3^-octreotide (40k cpm) with or without increasing concentrations of DOTATATE, ^115^In-DOTATATE, ^175^Lu-DOTATATE, or ^209^Bi-DOTATATE in HEPES buffer (10 mM HEPES, 5mM MgCl_2_ and 0.02 g/L bacitracin pH 7.6) containing 0.2% BSA. After incubation for 1 h, 1 mL HEPES (4°C) was added to stop the reaction. Non-bound radioligand was separated from the membrane-bound radioligand by centrifugation for 2 min at 10000 g. The remaining pellet was washed twice with ice-cold HEPES buffer and counted in a γ-counter (Wallac Wizard 3, Perkin Elmer, Groningen, the Netherlands) [21]. One-way ANOVA was used to calculate significant differences of IC_50_ values of DOTATATE, ^115^In-DOTATATE, ^175^Lu-DOTATATE, and ^209^Bi-DOTATATE.

### Internalisation as function of peptide amount in SSTR_2_ positive CA20948 cell line

0.5x10^6^ CA20948 cells were incubated with ^111^In-DOTATATE, ranging from 1 nM to 390 nM, for 1 h at 37°C. After incubation ^111^In-DOTATATE was removed from the cells by washing the cells twice with 1 mL ice-cold PBS. Ice-cold strip medium (1mL, HBSS containing 20 mM sodium acetate, pH 5) was added to the cells and incubated for 10 min to remove membrane-bound (*ƒ*_*mem*_) ^111^In-DOTATATE from the receptor. The strip medium was collected and 1 mL of ice-cold strip medium was added to the cells for 10 min incubation and collected again. Sodium hydroxide (1mL, 1M) was added to the cells to detach the cells from the well and to determine the radioactivity inside the cells, this is the internalized fraction (*ƒ*_*int*_). The *ƒ*_*int*_ was collected separately from the *ƒ*_*mem*_. The collected fractions were measured by the γ-counter. The results were plotted as % activity (%A) compared to total applied activity. To determine the non-specific binding, an excess of DOTATATE (1x10^-6^M) was added to ^111^In-DOTATATE and incubated for 1 h. The *ƒ*_*mem*_ and *ƒ*_*int*_ fractions were measured on the γ-counter. No internalization assays were performed with BON cell line due to the low expression of SSTR_2_ receptors. The specific binding of *ƒ*_*mem*_ and *ƒ*_*int*_ is the binding in 0.5x10^6^ CA20948 cells minus the non-specific binding. The specific binding for 0.5x10^6^ CA20948 cells were extrapolated to an uptake in 500 cells, these extrapolated uptakes were used to calculate the binding of 500 cells used for clonogenic assay. ^111^In-DOTATATE was only used to investigate the cell uptake kinetics, in this study potential cytotoxic effects caused by absorbed dose were neglected.

### Clonogenic assay

Survival curves of BON and CA20948 cells were determined by clonogenic assay after irradiation with ^137^Cs, ^177^Lu-DTPA, ^177^Lu-DOTATATE, ^213^Bi-DTPA or ^213^Bi-DOTATATE. Before irradiation of the cells, 500 cells were seeded in 6-well plates (pre-coated with poly-l-lysine) 24 h before the experiment. The cells were treated with increasing doses of radioligand, diluted in internalization medium (30 mM HEPES and 0.25% BSA), and incubated for 1 h at 37°C in a humidified atmosphere of 5% CO_2_. After irradiation, internalization medium containing radioligand was removed. Cells were washed twice with PBS and incubated with medium containing 10% FBS for 12 d. Every 2 or 3 d, medium was replaced by fresh culture medium. In order to be able to compare the cytotoxic effect of ^213^Bi-DOTATATE vs ^177^Lu-DOTATATE, the starting amount of labelled peptide was kept constant, therefore excess peptide was added to the ^177^Lu-DOTATATE to a start concentration of 390 nM. Similarly, clonogenic assay was performed with different doses of external irradiation with ^137^Cs (dose-rate of 0.6 Gy/min): 0.5, 1, 2, 4, 5, 6, 8 and 10 Gy.

After incubation over a period of 12 days at 37°C in a humidified atmosphere of 5% CO_2_, cells were washed twice with PBS, fixed with 1 mL 100% ethanol and stained with 1 mL hematoxylin. The 6-well plates containing colonies were scanned with a HP-scanner at 200 dpi. The colonies in each well were counted by a clonocounter [23]. The survival was plotted as a function of absorbed dose and fitted to linear-quadratic (LQ) or linear-exponential curves.

### Non-specific binding absorbed dose

**Fig 1:**
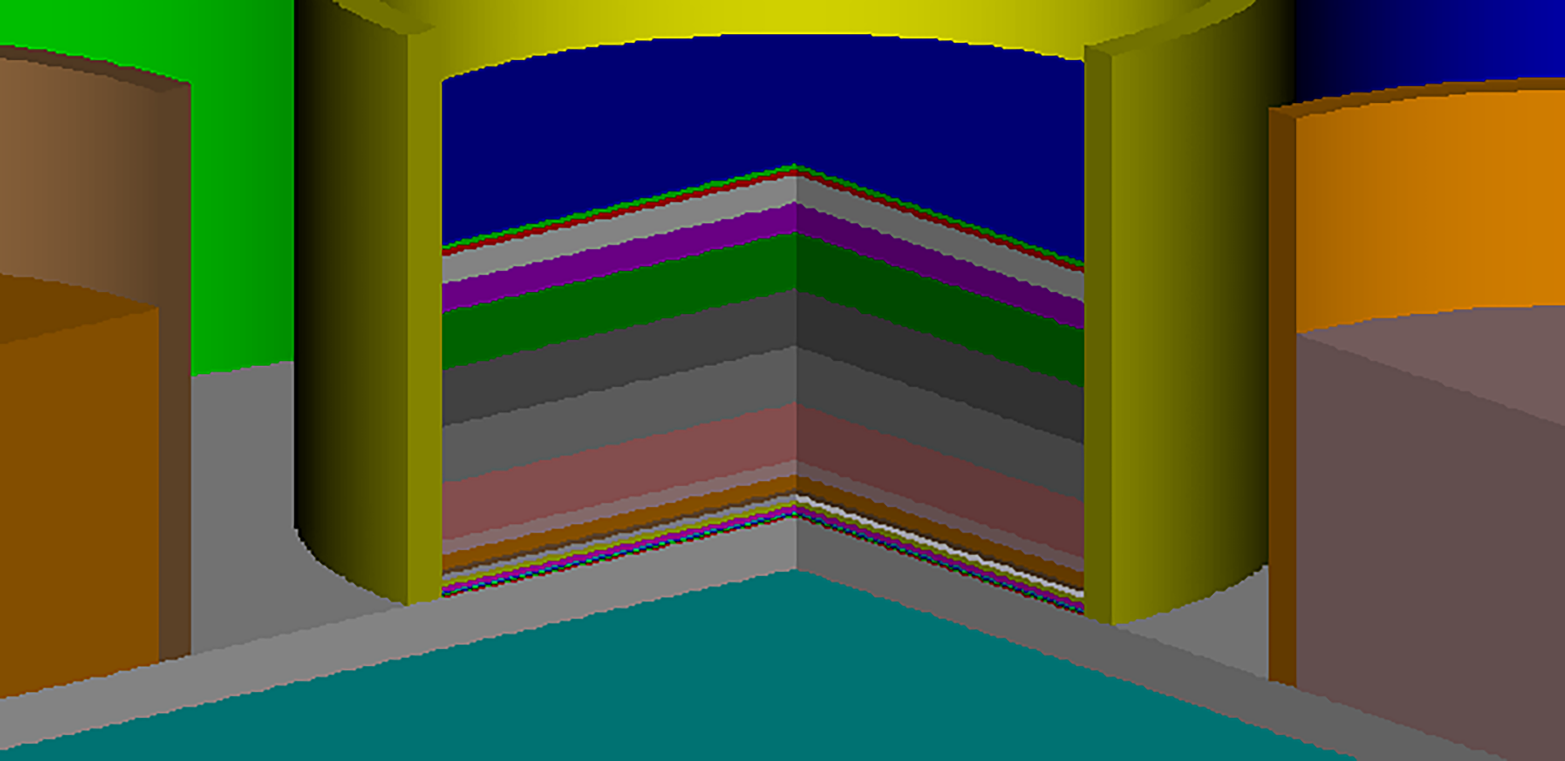
Illustration of layers (25 μm) divided over a total volume of 2 mL for the calculation of the absorbed dose in a 6-well plate.

The cell absorbed dose during the incubation period was calculated with a dosimetry model of the 6-well plate, as described by Verwijnen et al. [24]. The 3.5 cm diameter well cavity was modeled in the Monte Carlo radiation transport code Monte Carlo N-Particle eXtended MCNPX (version 2.5.0, Los Alamos National Laboratory, USA) with homogeneously distributed radioactivity over the 2 mL volume aqueous liquid within the cavity. Dose scoring regions of 25 μm were used to calculate the absorbed fractions of energy as a function of depth in the bottom 200 μm of the well, see Fig 1. The division the layers of the well were as followed; 18 layers of 100 μm and 8 layers of 25 μm at the bottom of the well.

The lowest layer of 25 μm was used for the calculation of absorbed dose at the bottom of the well containing 500 cells.

Radiation energy spectra for ^177^Lu, ^213^Bi and its daughters were derived from the MIRD radionuclide data handbook [25]. For each emission (α-, β- particles, low energy Auger- and internal conversion electrons and γ-rays) 10 million particle histories were used for particle transport calculations in MCNPX. Absorbed energy was calculated in the dose scoring regions within the cavity filled with radioactivity and the cross dose to the other 5 well cavities. Absorbed dose rates per unit activity (or S-values) for each radionuclide and dose scoring region were calculated following the MIRD schema [26].

The cells in the clonogenic assay were assumed to lie on the bottom surface of the 6-well plate cavities and therefore the macroscopic mean absorbed dose $\bar{D}$ to the cells during the 1 h incubation was calculated from the S-value in the bottom 25 μm layer and the time-integrated activity Ã by:

$$\bar{\text{D}}(25\mu m\leftarrow \mathrm{fluid})={Ã}_{\text{fluid}}(1)\times S(25\mu m\leftarrow \text{fluid})=\int_{{\text{T}}_{0}}^{\text{T}}{\text{A}}_{\text{fluid}}{\text{e}}^{\text{-}\lambda t}\text{dt}\times \text{S}(25\mu m\leftarrow \mathrm{fluid})$$

The integration was performed for the activity in the fluid from T_0_ = 0 to T = 1 h, also taking into account the progeny activity from ^213^Bi together with their specific S-values. The contributions to the absorbed dose by α-particles were considered separately from the dose by β-particles, low energy electrons and γ-rays.

### Specific binding absorbed dose

For cell dosimetry calculation, the total uptake (*ƒ*_*mem*_ and *ƒ*_*int*_) of 0.5x10^6^ cells (CA20948) was extrapolated to the uptake of 500 cells. The percentage of uptake was then used for cell dosimetry with the assumption that the radioactivity was bound to the cell membrane (*f*_*int*_ = 0 and *f*_*mem*_ = 1) over a period of 12 d without dissociation of the radioactivity.

The dosimetry for the specific uptake of radioactivity in the cells was calculated with the multicellular dosimetry code MIRD cell [27]. For the calculation of dosimetry in CA20948, a cell radius of 6 μm was taken, as the uptake in the cells was not further differentiated, only uptake on the cell surface and homogeneous cell uptake were considered. Initially 500 cells were plated over the 9.62 cm^2^ surface of the well. For evenly distributed cells over the surface the mean inter-cell distance would be 770 μm. In reality the cells were more clustered to the center of the well. Furthermore, the inter-cell distance was larger than the range of the α-particles and hence the cross-dose contribution from radioactivity taken up in neighboring cells was assumed to be minimal.

The number of disintegrations within each cell (or Time-Integrated Activity per cell ã) was calculated by integration of the decay function of ^177^Lu and ^213^Bi and its progeny over the 12 d irradiation time multiplied by the *ƒ*_*int*_ and *ƒ*_*mem*_ per cell. This assumes that *ƒ*_*mem*_ will remain bound to the cells and *ƒ*_*int*_ remains trapped in the cell over a period of 12 d. The cellular S values were obtained from the MIRD cell code for a cell with a radius of 6 μm and indicated in Table 1. The absorbed doses to the cell are indicated per decay of ^213^Bi and its daughters ^213^Po, ^209^Tl, ^209^Pb and for ^177^Lu in the cell or on the surface of a spherical cell with a radius of 6 μm. Also the cross-dose S-values for cells with radioactivity at 50 μm and at 100 μm are indicated.

**Table 1:** S-values (mGy/MBq.s) of ^213^Bi, ^213^Po, ^209^Tl, ^209^Pb and ^177^Lu for a cell with a radius of 6 μm according to MIRDcell code.

	Self-dose		Cross-dose S(C←C’)	
	S(C ← C)	S (C ← CS)	Cell distance 50 μm	Cell distance 100 μm
^**213**^**Bi**	1.71	1.14	7.02^−3^	3.03^−4^
^**213**^**Po**	49.2	33.0	0.40	OOR
^**209**^**Tl**	0.73	0.44	1.31^−3^	3.15^−4^
^**209**^**Pb**	0.34	0.22	1.61^−3^	3.46^−4^
^**213**^**Bi + progeny**	50.2	33.6	0.52	3.48^−2^
^**177**^**Lu**	0.67	0.42	2.00^−3^	4.18^−4^

OOR = out of range C = cell, C’ = other cell, CS = cell surface

The mean self-absorbed dose to the cells was calculated by the product of Ã_cell_ the mean cumulated activity per cell with the cellular S-values specific for the binding site:

$\bar{{\text{D}}_{\text{cell}}}={Ã}_{\text{cell}}[{\text{f}}_{\text{int}}\text{S}(\text{C}\leftarrow \text{C})+{\text{f}}_{\text{mem}}(\text{C}\leftarrow \text{CS})]$, with

$${Ã}_{\text{cell}}(\text{Bi-}213)=\int_{{\text{T}}_{0}}^{\text{T}}\bar{{\text{A}}_{\text{cell}}^{\text{Bi}}}\;{\text{e}}^{\text{-}\lambda_{\text{Bi}}\text{t}}\text{dt}$$

$${Ã}_{\text{cell}}(\text{Po-}213)=\int_{{\text{T}}_{0}}^{\text{T}}\bar{{\text{A}}_{\text{cell}}^{\text{Bi}}}\;\frac{{\text{BR}}_{\text{Po}}\lambda_{\text{Po}}}{\lambda_{\text{Bi}}\text{-}\lambda_{\text{Po}}}\left({{\text{e}}^{\text{-}\lambda_{\text{Po}}\text{t}}\text{-e}}^{\text{-}\lambda_{\text{Bi}}t}\right)\text{dt}$$

$${Ã}_{\text{cell}}(\text{Tl-}209)=\int_{{\text{T}}_{0}}^{\text{T}}\bar{{\text{A}}_{\text{cell}}^{\text{Bi}}}\;\frac{{\text{BR}}_{\text{Tl}}\lambda_{\text{Tl}}}{\lambda_{\text{Bi}}\text{-}\lambda_{\text{Tl}}}\left({{\text{e}}^{\text{-}\lambda_{\text{Tl}}\text{t}}\text{-e}}^{\text{-}\lambda_{\text{Bi}}\text{t}}\right)\text{dt}$$

$${Ã}_{\text{cell}}(\text{Pb-}209)=\int_{{\text{T}}_{0}}^{\text{T}}\bar{{\text{A}}_{\text{cell}}^{\text{Bi}}}\;\left\{\frac{{\text{BR}}_{\text{Po}}{\lambda_{\text{Po}}\lambda }_{\text{Pb}}}{\lambda_{\text{Bi}}\text{-}\lambda_{\text{Pb}}}(\frac{{{\text{e}}^{\text{-}\lambda_{\text{Pb}}\text{t}}\text{-e}}^{\text{-}\lambda_{\text{Bi}}\text{t}}}{\lambda_{\text{Bi}}\text{-}\lambda_{\text{Pb}}}+\frac{{{\text{e}}^{\text{-}\lambda_{\text{Po}}\text{t}}\text{-e}}^{\text{-}\lambda_{\text{Pb}}\text{t}}}{\lambda_{\text{Po}}\text{-}\lambda_{\text{Pb}}})\text{-}\frac{{\text{BR}}_{\text{Tl}}\lambda_{\text{Tl}}\lambda_{\text{Pb}}}{\lambda_{\text{Bi}}\text{-}\lambda_{\text{Tl}}}(\frac{{{\text{e}}^{\text{-}\lambda_{\text{Pb}}\text{t}}\text{-e}}^{\text{-}\lambda_{\text{Tl}}\text{t}}}{\lambda_{\text{Tl}}\text{-}\lambda_{\text{Pb}}}+\frac{{{\text{e}}^{\text{-}\lambda_{\text{Pb}}\text{t}}\text{-e}}^{\text{-}\lambda_{\text{Bi}}\text{t}}}{\lambda_{\text{Bi}}\text{-}\lambda_{\text{Pb}}})\right\}\text{dt}$$

The integration over time was performed between T_0_ = 1 h and T = 12 d. It was assumed that no radioligand cleared from the cells once it was bound. The absorbed dose originating from the cellular uptake during the 1 h incubation period was neglected.

## Results

### Labelling

The incorporation for all used radiolabelled peptide was >99% and the RCP of ^111^In-DOTATATE, ^177^Lu-DOTATATE and ^213^Bi-DOTATATE were >95%, >95%, and >85%, respectively. The RCP of ^125^I-Tyr^3^-octreotide was >99%. The chemical yield of ^115^In-DOTATATE, ^175^Lu-DOTATATE and ^209^Bi-DOTATATE were >99%.

### Affinity study and internalization

Under the conditions applied, DOTATATE, ^115^In-DOTATATE, ^175^Lu-DOTATATE and ^209^Bi-DOTATATE showed similar affinities to SSTR_2_ on the membranes of K562-SST_2_ cells. IC_50_ values of 3.0±0.9 nM, 2.5±0.4 nM, 2.7±0.4 nM and 5.2±1.0 nM were found, respectively. No significant differences were found between the IC_50_ values.

### Internalization in SSTR_2_ positive CA20948 cell line

**Fig 2:**
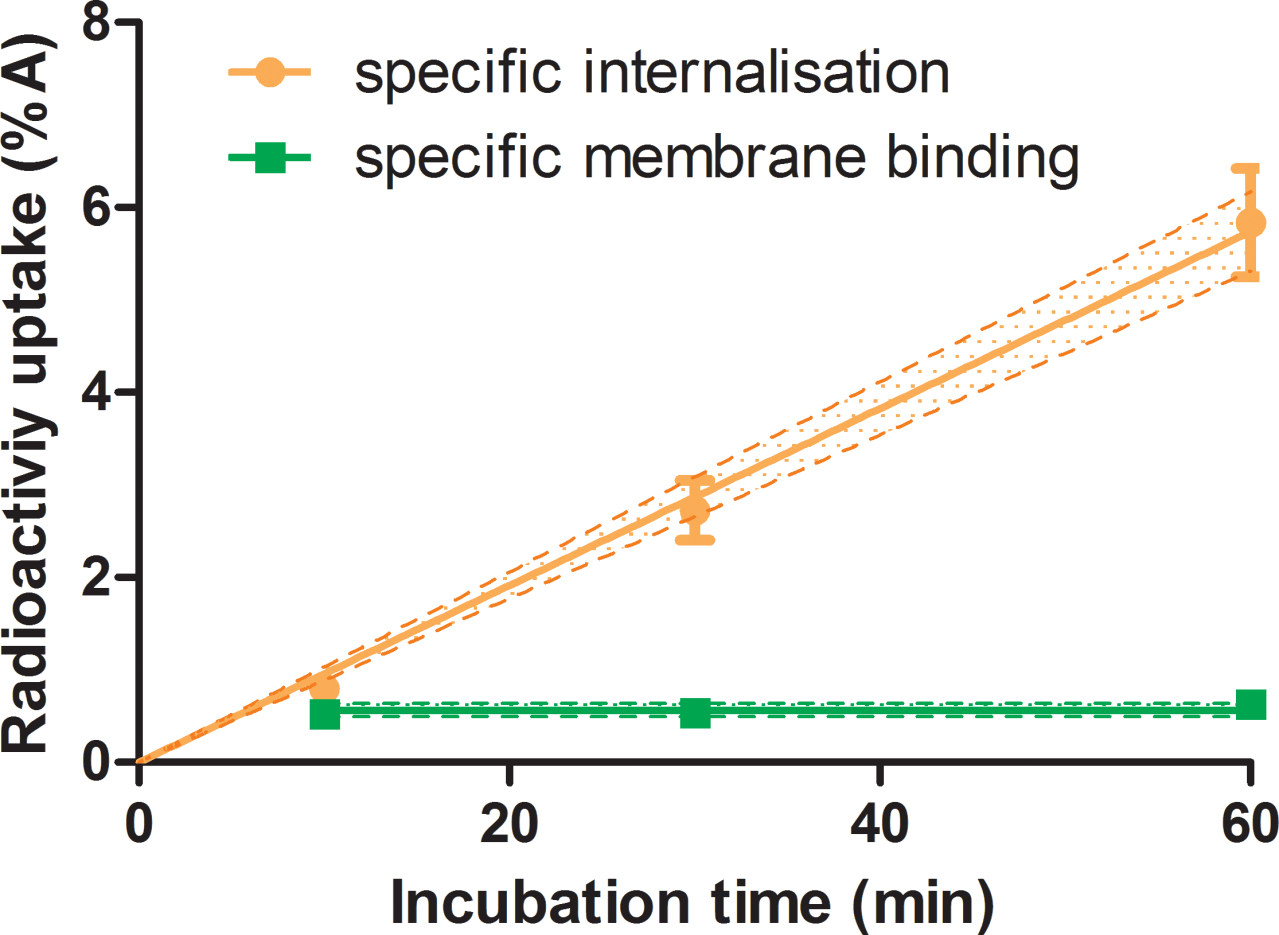
Specific membrane binding and specific internalization of 1 nM ^111^In-DOTATATE on 0.5x10^6^ cells of CA20948 as function of incubation time, n = 3.

Internalization was performed to determine the amount of radioligand bound to the receptors, at the cell membrane and internalized into the cell. Optimized concentration of ^111^In-DOTATATE for internalization on CA20948 was 1 nM. At this concentration, the *f*_int_ increased linearly as a function of incubation time. However, the *f*_mem_ remained constant at a level of 0.008%A as a function of incubation time, see Fig 2.

The lines indicate the fitted curves with the 95% confidence intervals, internalization data linear curve (R^2^ = 0.97) with slope: 0.096 ± 0.003%IA/min and for the *f*_mem_ the mean value of 0.56 ± 0.06%IA.

**Fig 3:**
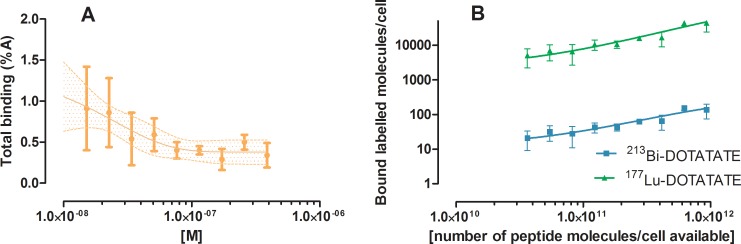
**A) Total binding of 1.5x10**^**-8**^ **to 3.9x10**^**-7**^ **M** ^**111**^**In-DOTATATE with 0.5 million CA20948 cells after 60 min incubation (n = 3) and B) the number of labelled** ^**213**^**Bi-DOTATATE or** ^**177**^**Lu-DOTATATE molecules bound per cell (y-axis) versus the total number of peptide present per cell (x-axis) during clonogenic assay.** The line and shaded area in Fig 3A indicate the fit (R^2^ = 0.4) and its 95% confidence interval of a single exponential curve *Ae*^*-k[M]*^ *+ B* with A = 1.0±0.5%A, k = 39±24 μmol^-1^ and B = 0.38±0.07%A.

Binding assays with ^213^Bi-DOTATATE involved high concentrations of unlabelled peptide due to the low molar activity of labelled peptide. At the highest peptide amount used for the experiment, (390 nM), an equilibrium of association and dissociation was found within 30 min for *ƒ*_*mem*_ and 10 min for *f*_*int*_. Here we mimicked the internalization of ^213^Bi-DOTATATE and ^177^Lu-DOTATATE by using ^111^In-DOTATATE as a surrogate to study the uptake kinetics with the concentration used in the clonogenic assay. Cells were incubated with decreasing concentrations of peptide (ranging 390 to 15 nM) for 1 h. The uptake was found to be relatively low, the *ƒ*_*mem*_ and *ƒ*_*int*_ were difficult to distinguish from each other, and therefore the sum of *ƒ*_*mem*_ and *ƒ*_*int*_ was used for further cell dosimetry calculations, see Fig 3A. The uptake was used to calculate the number labelled peptide, ^213^Bi-DOTATATE or ^177^Lu-DOTATATE, bound per cells during clonogenic assay with ^213^Bi-DOTATATE and ^177^Lu-DOTATATE in CA20948, see Fig 3B.

### Clonogenic assay of BON and CA20948 cells using external irradiation

**Fig 4:**
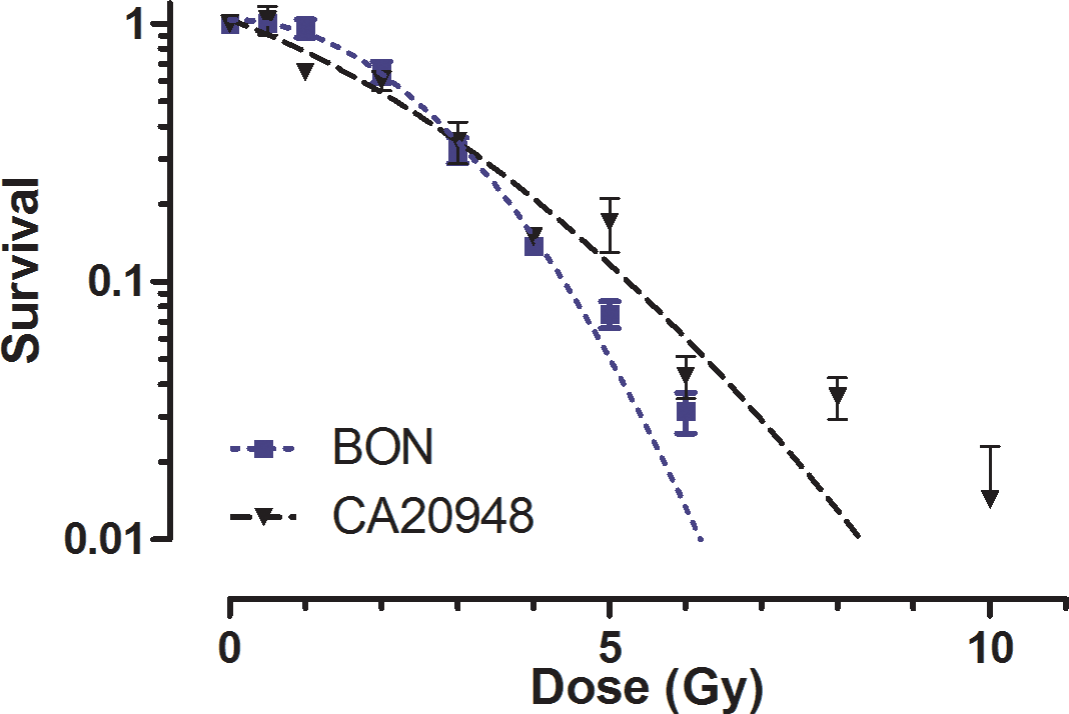
Survival curves of BON and CA20948 cells obtained after exposure at different doses of ^137^Cs γ-radiation, n = 3. The x-axis is expressed in dose (Gy) and the y-axis in survival fraction. Each data point is plotted as the mean survival ± SD. The curves through the data lead to the following LQ model parameters; BON: α < 0.11 Gy^-1^, β = 0.12±0.02 Gy^-2^ and CA20948: α = 0.21±0.07 Gy^-1^, β = 0.05±0.02 Gy^-2^.

The survival of BON and CA20948 cells after exposure to escalating absorbed doses with a ^137^Cs source is showed in Fig 4. Up to absorbed doses of 4 Gy similar survival curves were found for both cell lines. At absorbed doses above 4 Gy, CA20948 showed to be more radio-resistant than BON, see Table 2. Curves according to the LQ model were fitted with high correlation coefficients (R^2^ = 0.98 and 0.93, respectively), indicating that the curvature of the BON cells was more profound than for CA20948.

**Table 2:** Survival fraction of BON and CA20948 cells after exposure of ^137^Cs at 2, 4, 6 and 8 Gy.

Cell line	SF2	SF4	SF6	SF8
**BON**	0.65±0.10	0.14±0.016	0.031±0.010	0.07±0.002
**CA20948**	0.60±0.09	0.15±0.013	0.043±0.014	0.036±0.014

### Dosimetry

The time-integrated activity coefficient in the well cavity during the 1 h incubation period was 2361 MBq.s per MBq ^213^Bi, leading to an absorbed dose of 1.60 Gy/MBq in the 2 mL fluid and 1.12 Gy/MBq in the bottom 25 μm layer. The absorbed dose was delivered 96.2% by the α-particles, see Table 3.

**Table 3:** Absorbed fraction of energy ϕ and absorbed dose rate per MBq radioactivity ^213^Bi and its daughters calculated in the bottom 25 μm of the 2 mL fluid, which contributed to the calculated absorbed dose.

	^213^Bi ϕ (%)	S (25μm←well) (mGy/MBq.s)	^213^Po ϕ (%)	S (25μm←well) (mGy/MBq.s)	^209^Tl ϕ (%)	S (25μm←well) (mGy/MBq.s)	^209^Pb ϕ (%)	S (25μm←well) (mGy/MBq.s)
**α**	1.00	0.0082	0.82	0.4570				
**β**	0.56	0.0159			0.50	0.0218	0.64	0.0084
**Auger/IC**	0.61	0.0008			0.72	0.0015		
**γ**	0.03	0.0001			0.01	0.0010		
**Total**		0.0249		0.4570		0.0243		0.0084

### Clonogenic assay of BON and CA20948 cells using radiolabelled ligand

BON and CA20948 cells were treated with increasing amounts of radioactivity coupled to the radioligand; ^213^Bi-DTPA, ^213^Bi-DOTATATE, ^177^Lu-DTPA and ^177^Lu-DOTATATE. The BON cells showed increased cytotoxic effect at increasing radioactivity of ^213^Bi-DTPA and ^213^Bi-DOTATATE.

**Fig 5:**
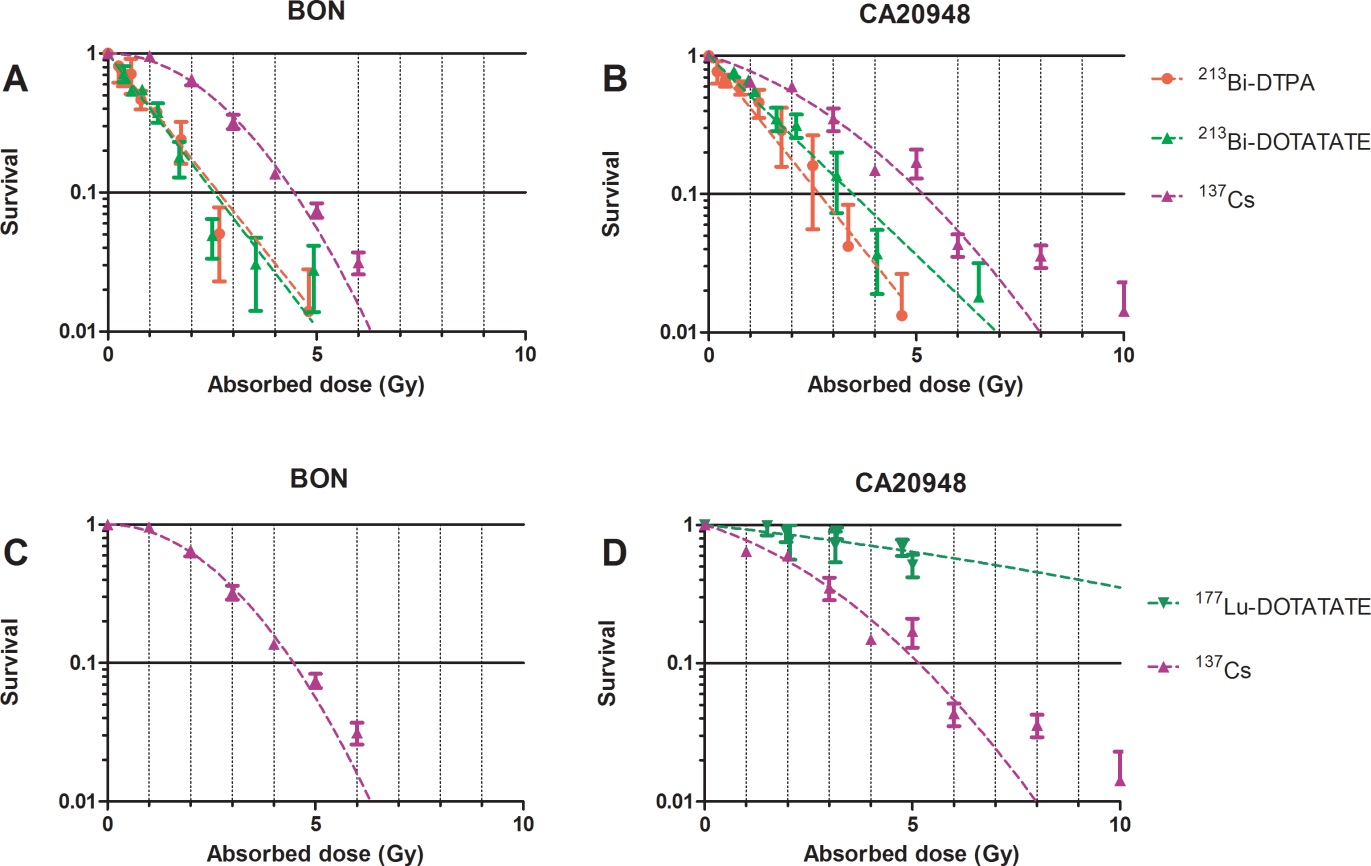
Survival curves of BON and CA20948 after exposure of ^137^Cs γ-radiation, ^177^Lu-DOTATATE, ^213^Bi-DTPA, and ^213^Bi-DOTATATE, n = 3. The x-axis is expressed in absorbed dose to the cells (Gy) and the y-axis in percentage survival. Each data point is plotted as the mean survival ± SEM. Cells were incubated with ^177^Lu-DOTATATE, ^213^Bi-DOTATATE and ^213^Bi-DTPA for 1h. Cells were fixed and colonies were measured 12 d after treatment.

In BON cells, similar survival curves were found after irradiation with ^213^Bi-DOTATATE or ^213^Bi-DTPA. No significant differences were observed in the slopes of the survival curves (Fig 5A). The α/β ratio obtained from the linear-quadratic model describing irradiation by ^137^Cs was found to be <0.01 Gy for BON and 4.5±3.6 Gy for CA20948. The survival curves of BON and CA20948 treated with non-specific binding of ^213^Bi-ligand fitted with a one-phase decay model. In CA20948 cells, a decreased survival at increased radioactivity of ^213^Bi-radioligand was observed. No significant differences in survival were observed between ^213^Bi-DOTATATE and ^213^Bi-DTPA, see Fig 5B.

No reduction of BON cell survival was found after treatment with ^177^Lu-DOTATATE for 1 h. A very low binding was present, resulting in low calculated dose, <1Gy (Fig 5C). As for irradiation of CA20948 cells with ^177^Lu-DTPA, cell survival was found to be around 100% after irradiation with > 6 MBq ^177^Lu-DTPA for 1 h (data not plotted). In case of ^177^Lu-DOTATATE, a reduction of cell survival was found with increased radioactivity, see Fig 5D.

The LQ model parameters α, β and α/β calculated after ^137^Cs, ^213^Bi-DTPA, ^213^Bi-DOTATATE, ^177^Lu-DOTATATE irradiation for BON and CA20948 are shown in Table 4.

**Table 4:** The LQ model parameters α, β and α/β calculated for BON and CA20948.

	BON			CA20948		
	α(/Gy)	β(/Gy^2^)	α/β(Gy)	α(/Gy)	β(/Gy^2^)	α/β(Gy)
^**137**^**Cs**	< 0.11	0.12±0.02	0	0.21±0.07	0.05±0.02	4.5±3.6
^**213**^**Bi-DTPA**	0.87±0.1	-	-	0.74±0.08	-	-
^**213**^**Bi-DOTATATE**	0.91±0.05	-	-	0.56±0.03	-	-
^**177**^**Lu-DOTATATE**	-	-	-	0.08±0.03	0.003±0.004	25.2±37.8

## Discussion

In this study we selected DOTATATE as a targeting ligand. To investigate the distribution, time-dependent and peptide amount-dependent uptake, DOTATATE was labelled with the γ-emitting radionuclide ^111^In. The results obtained were used for further cell averaged dosimetry. The effect on cell survival caused by radiation was compared using β-emitting ^177^Lu-DOTATATE and α-emitting ^213^Bi-DOTATATE. Cellular uptake is an important and critical parameter for the success of peptide receptor radionuclide therapy due to receptor-mediated process, therefore a high and a low SSTR_2_ expression cell line were chosen here, to compare the receptor-dependent-survival. Cell averaged dosimetry was performed. The activity uptake in the cells was analyzed for 500 cells and the high LET character of the individual α-particle tracks were averaged out for dosimetry calculation.

^111^In-DOTATATE, ^177^Lu-DOTATATE, ^213^Bi-DOTATATE and DOTATATE were shown to have similar affinity for SSTR_2_. IC_50_ values were similar of labelled peptides i.e. in the nM range. Cellular uptake of 1nM ^111^In-DOTATATE demonstrated an increase of internalization in cells with increase of incubation time, compared to the time-independent specific membrane bound fraction *f*_mem_ in CA20948. In this study the specific binding and internalization of 390 nM peptide as function of incubation time was also investigated. Since the MA of ^213^Bi-DOTATATE obtained after labelling with a ^213^Bi/^225^Ac generator of 222 MBq was low [18], a concentration of 390 nM labelled peptide was required for the sufficiently high radioactivities (approximately 4 MBq) in the clonogenic assays. To mimic the peptide concentrations used for further clonogenic assays, peptide-amount dependent uptakes resulting from incubation with 15 to 390 nM ^111^In-DOTATATE were investigated. Very low *ƒ*_*mem*_ and *ƒ*_*int*_ were found in this range of concentrations, see Fig 3A. Based on the activity measured of ^177^Lu-DOTATATE or ^213^Bi-DOTATATE, the data obtained from peptide-amount dependent uptake with ^111^In-DOTATATE, extrapolated to 500 cells and taking the radionuclides’ MA into account, about 150±30 ^213^Bi-DOTATATE molecules were bound per cell with the highest radioactivity amount used for clonogenic assay. This is low in comparison to the 44000±8000 ^177^Lu-DOTATATE molecules at the same peptide amount, but still high enough to warrant average cellular dosimetry.

In this study we found that the highest cytotoxic effect was caused by ^213^Bi-ligands. The theoretical advantage of using α-emitters as therapeutic agents is the independence of dose rate, oxygenation and cell proliferation [28, 29]. Cytotoxic effects caused by ^213^Bi-DOTATATE demonstrated no discrimination between cell types and level of SSTR_2_ expression. We observed a similar cytotoxic effect with ^213^Bi-DTPA and ^213^Bi-DOTATATE in CA20948, indicating a cytotoxic dose can be delivered to the cells in vitro without specific binding and peptide receptor-mediated endocytosis. The low MA of the labelled peptide led to rapid saturation of the receptors by unlabelled peptides thereby causing a higher concentration of labelled peptide in the incubation medium. The effect and absorbed dose by the specific binding was therefore reduced in comparison to the contribution by the medium. The absorbed dose during incubation of the cells, which is independent of specific uptake, forms the main contribution to the total dose, 53–76% depending on the amount of activity. The absorbed dose by the specific binding fraction can be increased to levels similar to the dose contribution from the medium exposure by using activities > 10 MBq. This would correspond to a minimal requirement of a ^225^Ac generator twice the activity used in the current experiments (222 MBq). Furthermore, the irradiation time can then be reduced to 30 min.

In our study the *D*_10_ in BON was 4.5 Gy for ^137^Cs, 2.5 Gy with ^213^Bi-DTPA and 2.6 Gy with ^213^Bi-DOTATATE. In CA20948, *D*_10_ values observed with ^137^Cs, ^213^Bi-DOTATATE and ^213^Bi-DTPA in CA20948 were 5.1, 3.3 and 2.6 Gy, respectively. Due to the low uptake at low dose of ^213^Bi-DOTATATE, heterogeneous distribution of ^213^Bi-DOTATATE, MA (Bq/nmol) on the cell played a significant role here, the number of DNA hits appeared to be insufficient to influence cell survival. Heterogeneous distribution can influence the amount of ionizations traversing to the DNA of the cell causing irreparable DBS, as described by Pasternak et al (9). The advantage of increasing the MA of labelled antibodies by an antibody cocktail caused a more homogenous binding to the cells and increased the cell-killing ability of α-emitters [9].

In the case of ^177^Lu-ligand, peptide receptor-mediated endocytosis is essential to cause cytotoxic effect in the cells. Due to the low LET of β-emitters, at least 1000–4000 β-particles are required to lead to non-dividing cells and cell death. BON cells, with low SSTR_2_ expression, showed no reduction in cell survival after irradiation with ^177^Lu-DOTATATE. In CA20948 cells survival showed a correlation with absorbed dose using ^177^Lu-DOTATATE, a *D*_10_ of 17.8 Gy was found after extrapolation of the LQ model fit to *D*_10_. For both cell lines, ^177^Lu-DTPA showed no effect on cell survival. We found that the absorbed dose was low for ^177^Lu-DTPA in both cell lines and for ^177^Lu-DOTATATE in BON, <0.2 Gy. Therefore, *D*_10_ cannot be determined. Specific binding of ^177^Lu-DOTATATE (0.33 MBq, lowest radioactivity used to treat cells) to CA20948 resulted in an absorbed dose at least 7 times higher than without specific binding (^177^Lu-DTPA).

In our study, only 500 cells were plated in the well for the clonogenic assay and were assumed to be spherical in shape with a diameter of 12 μm and homogenously distributed on the bottom of the well. The average distance between homogeneously distributed cells was approximately 1500 μm. The maximum path length in tissue for α-particle is 50–100 μm and for β-particle is 1–10 mm. Absorbed dose by cross-fire contributed from ^177^Lu after incubation with ^177^Lu-DOTATATE can be neglected, since the maximum path length of ^177^Lu in tissue is approximately 2000 μm [30], the dose caused from cross-fire effect of ^177^Lu was less than 1x10^-12^ Gy with a distance of the 1500 μm [31]. As for ^213^Bi-DOTATATE, the maximum path length of ^213^Bi is 80 μm and the distance between the cells was >1500 μm, an additional dose to the calculated absorbed dose caused by cross-fire effect was not taken into account. Despite this we assumed that the cells were homogeneous distributed, as clustering of cells can still occur, leading to an increase of the absorbed dose caused by cross-fire effect of neighboring cells. This was often observed in studies using low energy β-particles such as ^177^Lu [31]. Therefore, this might have resulted in an underestimation of the absorbed dose caused by ^177^Lu in our calculations. The bound activity was assumed to remain on the cells during the 12 d irradiation, as externalization was excluded. This assumption will have no effect on the absorbed dose by ^213^Bi-DOTATATE, since the half-life of ^213^Bi is short, 90% of the dose was delivered to the cells within 3.5 h. For ^177^Lu-DOTATATE, the calculated absorbed dose will consequently be an overestimation the actual absorbed dose.

The calculated RBE at *D*_10_ with ^213^Bi-DTPA compared to ^137^Cs irradiation was 2.0 in CA20948 and 1.8 in BON. As for ^213^Bi-DOTATATE compared to ^137^Cs in CA20948 and BON, the RBE was found to be 1.5 and 1.7, respectively. At *D*_20_, Nayak and co-workers found ^213^Bi-DOTATOC to be 3.4 times more cytotoxic than ^177^Lu-DOTATOC in terms of RBE in CAPAN-2 cell line (human pancreatic adenocarcinoma) [7]. In our study we also found that ^213^Bi-DOTATATE was more potent for cell killing than ^177^Lu-DOTATATE. The amount of ^213^Bi-DOTATATE molecules bound per cell was a factor of approximately 300 less than ^177^Lu-DOTATATE molecules bound per cell. RBE’s of 5.4 at *D*_10_ and 5.7 at *D*_20_ were found for ^213^Bi-DOTATATE in comparison to ^177^Lu-DOTATATE. The RBE was higher compared to that found in the study of Nayak et al., this was probably caused by the high dose contributed from the internalization medium in our study. Graf et al. demonstrated the high cytotoxic effect caused by α-emitters using ^225^Ac-DOTATOC in rat pancreatic carcinoma cell line, AR42J [6]. An ED_50_ of 14 kBq/mL was found for ^225^Ac-DOTATOC and 10 MBq/mL for ^177^Lu-DOTATOC. Higher amounts of γH2AX (biomarker of DSB) were observed in cells treated with ^225^Ac-DOTATOC. They found a comparative cytotoxicity assessment by a factor approximately of 700 between ^177^Lu and ^225^Ac at ED_50_. In our study, we found a factor of approximately 5.7 between ^213^Bi-DOTATATE (0.33 MBq/mL) and ^177^Lu-DOTATATE at ED_50_ (1.88 MBq/mL). The large difference was caused by the 4 α-particles release of ^225^Ac compared to one α-particles release of ^213^Bi and the exposure time; 48 h versus 1 h.

Radionuclide therapy with ^213^Bi-DOTATATE showed to be capable of treating both small metastasis and observable large tumours [5]. Cell uptake for targeted radionuclide therapy is an essential factor for the calculation of absorbed dose in TAT, as well as selected endpoints and experimental design. Absorbed dose calculation methods used in this study described the average absorbed dose caused by TAT with ^213^Bi, enabling comparisons between different cell irradiation experiments on basis of average absorbed doses at *D*_10_.

## Conclusion

^213^Bi-DTPA and ^213^Bi-DOTATATE showed higher cytotoxic effects than ^177^Lu-DTPA and ^177^Lu-DOTATATE in highly SSTR_2_ expressing cells. RBE’s at 10% cell survival ranging from 1.5–2.0 were found for ^213^Bi-DTPA and ^213^Bi-DOTATATE in both low and high SSTR_2_ expressing cell lines under the conditions applied. Cellular dosimetry calculations allow comparisons to be made between α-, β-emitters and external γ-radiation sources. Per Gy delivered ^213^Bi-DOTATATE was at least 5 times more effective in cell killing in comparison to ^177^Lu-DOTATATE.
